# Risk of Diabetes-Specific Eating Disorders in Children with Type 1 Diabetes Mellitus Using Continuous Subcutaneous Insulin Infusion: A CGM-Based Cross-Sectional Study

**DOI:** 10.3390/medicina61091585

**Published:** 2025-09-02

**Authors:** Özge Köprülü, Hülya Tan, İbrahim Mert Erbaş, Fatma Yavuzyılmaz Şimşek, Nilüfer Uyar, Murat Çağlar Karataş, Özlem Nalbantoğlu, Hüseyin Anıl Korkmaz, Behzat Özkan

**Affiliations:** 1 Department of Pediatric Endocrinology, Dr. Behçet Uz Children’s Education and Research Hospital, 35210 Izmir, Turkeyibrahimmert.erbas@sbu.edu (İ.M.E.); ozlem.nalbantoglu@sbu.edu (Ö.N.); huseyinanil.korkmaz@sbu.edu (H.A.K.); behzat.ozkan@sbu.edu (B.Ö.); 2 Department of Pediatrics, İzmir Faculty of Medicine, University of Health Sciences, 35210 Izmir, Turkey

**Keywords:** diabetes-specific eating disorders, DEPS-R, continuous subcutaneous insulin infusion, continuous glucose monitoring, time in normoglycemia

## Abstract

*Background and Objectives*: Eating disorders are one of the most widespread health concerns, mainly among adolescents. Children and adolescents with type 1 diabetes mellitus (T1DM) have been reported to have a high prevalence of eating disorders. The aim of our study is to evaluate the risk of diabetes-specific eating disorders in children with T1DM using continuous subcutaneous insulin infusion (CSII), with real-time glycemic data from continuous glucose monitoring (CGM). *Materials and Methods*: Sixty-four patients (aged 7–18 years) completed the Diabetes Eating Problem Survey-Revised (DEPS-R). The DEPS-R is a diabetes-specific self-report questionnaire to assess diabetes-specific compensatory behaviors. Auxological findings, sex, age, age at diagnosis, hemoglobin A1c (HbA1c) levels, and all CGM data were obtained from their medical records. *Results*: Although the median DEPS-R score was higher in children and adolescents using CSII compared to those using multiple daily injections (MDIs) (14 vs. 11), the difference was not statistically significant (*p* = 0.302). The risk of diabetes-specific eating disorders was identified in six patients (30%) using CSII and in nine patients (20.4%) using multiple daily injections (*p* = 0.403). Interestingly, in the subgroup with poor glycemic control (HbA1c > 9%), DEPS-R scores were significantly lower among those using CSII compared to the MDI group. Pearson correlation analysis demonstrated positive associations between DEPS-R scores and diabetes duration, weight SDS, body mass index (BMI), BMI SDS, HbA1c, mean glucose, Glucose Management Indicator (GMI), time above range (TAR) (very high), and coefficient of variation (CV), while a moderate negative correlation was observed with time in range (TIR). *Conclusions*: This study showed that the treatment of CSII had a beneficial effect on the risk of eating disorders in patients with poor glycemic control. As well, from this perspective, CSII maintains its status as a potentially beneficial therapeutic approach in diabetes management.

## 1. Introduction

Eating disorders are characterized by a persistent disturbance of eating or eating-related behavior that results in the altered consumption of food that impairs physical health or psychosocial functioning [1].

Eating disorders are one of the most widespread health concerns, mainly among adolescents. The etiology remains unclear, and management is challenging, as some patients do not respond to any available treatments. Females are more likely to be affected by this disease [2]. Likewise, patients with type 1 diabetes mellitus (T1DM) have been reported to have a high prevalence of eating disorders [3,4,5,6].

To date, an increasing number of studies have focused on the association of diabetes-specific eating disorders in children and adolescents with T1DM. Eating disorders in adolescents with type 1 diabetes mellitus (T1DM) are of significant clinical importance for glycemic control [6]. Poorer glycemic control, a history of dietary restraint, increased body mass index (BMI), female gender, the focus on diet and carbohydrate intake, risk of insulin-related weight gain and associated body dissatisfaction, and emotional dysregulation are the main risk factors for eating disorders in adolescents with T1DM [5,6].

In order to evaluate children diagnosed with T1DM in terms of eating disorders, the DEPS-R questionnaire has been developed by Markowitz et al. [7] and its Turkish validity and reliability study has been carried out by Altınok et al. [8] in 2017.

Compared to multiple daily injections, continuous subcutaneous insulin infusion (CSII) is associated with lower glycated hemoglobin A1c (HbA1c) levels, fewer severe hypoglycemic episodes, and improved quality of life and treatment satisfaction in people with T1DM [9]. While the continuous subcutaneous insulin infusion provides significant advantages in diabetes treatment, it may lead to eating disorders [10]. Transition from restricted eating to flexible meal patterns, negative body image, weight gain, psychosocial risk, and hunger and satiety dysregulation appear to be important factors contributing to reports of diabetes-specific eating disorders in children with T1DM using continuous subcutaneous insulin infusion [11]. On the other hand, providing the greatest flexibility, continuous subcutaneous insulin infusion may be beneficial in eating disorders [12]. The aim of our study is to evaluate the risk of diabetes-specific eating disorders in children with T1DM using CSII, incorporating real-time glycemic data from CGM. By integrating behavioral assessments with objective glucose metrics, including mean glucose, standard deviation (SD), coefficient of variation (%CV), time in range, time in normoglycemia, time above range, and time below range, it provides an understanding of the potential protective role of CSII therapy against disordered eating behaviors.

## 2. Materials and Methods

This research was planned as a cross-sectional study. The study protocol was approved by the Dr. Behcet Uz Children’s Hospital Ethics Committee (2025/05-04) in accordance with the Helsinki Declaration. Detailed information about this study was given to children who were voluntary participants in this study and written informed consent was obtained.

The study group included 13 female and 7 male patients (*n* = 20) who were followed up for T1DM and using CSII. The control group (*n* = 44) consisted of age and sex-matched peers who were using multiple daily insulin injections (MDIs).

Exclusion criteria for both groups included presence of intellectual disability, any neurological disorder, psychotic disorder, and autism spectrum disorders. Individuals with any chronic and/or severe medical illness or complications of diabetes were also excluded.

Auxological findings, sex, age, age at diagnosis, and HbA1c levels were obtained from their medical records. All CGM data were reviewed, including mean glucose, standard deviation (SD), coefficient of variation (CV), Glucose Management Indicator (GMI); time in range (TIR; 70–180 mg/dL; 3.9–10 mmol/L), time above range (TAR; >10 mmol; >180 mg/dL), and time below range (TBR; <3.9 mmol/L; <70 mg/dL). Time above range (TAR) was categorized as high for glucose levels >180 mg/dL (>10 mmol/L) and very high for levels >250 mg/dL (>13.8 mmol/L); time below range (TBR) was categorized as low for glucose levels < 70 mg/dL (<3.9 mmol/L) and very low for levels < 54 mg/dL (<3 mmol/L). We also reviewed time in normoglycemia (TING; 63–140 mg/dL; 3.5–7.7 mmol/L), a relatively new metric that has recently begun to be used in pediatric studies to better capture near-normal glucose levels. Only records in which the sensor was worn for ≥70% of the last 14 days were included in our analyses. Participants who did not meet this duration or data completeness threshold were excluded from the main analyses and considered only in exploratory analyses.

Sixty-four patients (aged 7–18 years) completed the Diabetes Eating Problem Survey-Revised (DEPS-R). The DEPS-R is a diabetes-specific self-report questionnaire to assess diabetes-specific compensatory behaviors. The DEPS-R consists of 16 items, and answers are rated on a six-point Likert scale ranging from 0 (never) to 5 (always), with a cutoff score of 20 points indicating a high risk for eating disorders. This questionnaire was revised in 2010 by Markowitz et al. [7] and the reliability and validity of the Turkish version of the DEPS-R were confirmed by Altınok et al. [8]. The original DEPS-R has demonstrated strong internal consistency (Cronbach’s alpha = 0.86) and construct validity in children and adolescents with T1DM.

SPSS 25.0 (IBM Corporation, Armonk, New York, NY, USA) was used to analyze the variables. Due to the relatively small sample size, the assumption of normality was assessed not only with the Shapiro–Wilk test but also by visual inspection of histograms and Q-Q plots, which provide more sensitive information in small samples. Parametric statistical tests were used when the assumption of normality was met. Non-parametric statistical tests were used when the assumption was not normal. The Student’s *t*-test or the Mann–Whitney U test were used to assess differences between two groups based on the normal distribution of the parameters. The Chi-square test was used to compare the categorical variables. Quantitative variables were expressed as the mean (standard deviation) or the median (25th and 75th percentiles), and categorical variables were number (N) and frequency (%). The variables were analyzed at a 95% confidence level, and a *p*-value less than 0.05 was considered significant.

## 3. Results

The CSII group (mean age: 13.14 ± 3.67 years) and MDI (mean age: 13.42 ± 2.69 years) were similar with respect to age (*p* = 0.455). A total of 65% of the CSII group (*n*:13) and 52.2% of the MDI group (*n* = 23) were female (*p* = 0.341). Auxologic data were similar between the groups. Participants (*n*:64) had a median duration of T1DM of 3.08 (1.85–5.02) years and there was no difference between the groups. The auxologic and clinical characteristics of CSII and MDI group are summarized in Table 1.

Although the median DEPS-R score was higher in children and adolescents using CSII compared to those on MDI (14 vs. 11), the difference was not statistically significant (*p* = 0.302). The risk of diabetes-specific eating disorders was identified in six patients (30%) using insulin infusion pumps and in nine patients (20.4%) using multiple daily injections (*p* = 0.403). Interestingly, in the subgroup of patients with poor glycemic control (HbA1c > 9%), DEPS-R scores were significantly lower among those using continuous subcutaneous insulin infusion (CSII) compared to those not using CSII (Table 2).

Table 3 presents a comparison of sex, age, diabetes duration, BMI Z-scores, and HbA1c levels in children with type 1 diabetes mellitus, stratified by DEPS-R score categories (<20 and ≥20) and insulin therapy type. No significant differences were found between groups in terms of age, BMI Z-score, or diabetes duration across DEPS-R categories.

**Figure 1:**
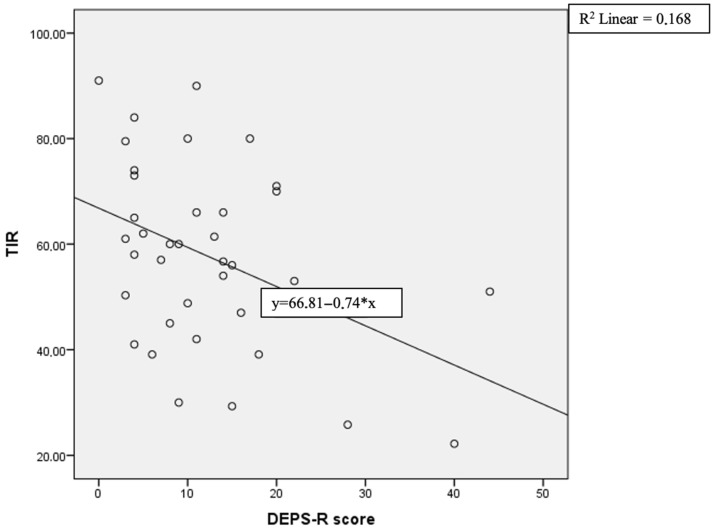
The negative correlation between TIR (time in range) and DEPS-R score (Diabetes Eating Problem Survey-Revised score). Each circle represents an individual patient data point. The (*) in the equation denotes multiplication.

As shown in Table 4, Pearson correlation analysis did not demonstrate any significant correlations between DEPS-R scores and age, age at diagnosis, weight, TING, TAR (high), TBR (low), TBR (very low), or SD. However, a weak but statistically significant positive association was observed between DEPS-R scores and both diabetes duration and weight SDS. Moreover, moderate positive correlations were found with BMI, BMI SDS, HbA1c, mean glucose, GMI, TAR (very high), and CV, while a moderate negative correlation was identified with TIR (Figure 1).

## 4. Discussion

In this study, we investigated the relationship between diabetes-specific eating disorder risk, as measured by the DEPS-R, and insulin therapy methods. The DEPS-R is a validated tool designed to detect disordered eating behaviors, specifically within the context of diabetes.

No significant difference was found in the risk of diabetes-specific eating disorders (as measured by the DEPS-R) between children with T1DM using CSII and those using MDI. Similarly, DEPS-R scores were similar between the patients using MDI and CSII to those reported in previous studies [5,8,13,14,15,16,17,18,19]. Our findings contrast with data from certain studies suggesting reduced risk of CSII therapy on diabetes-specific eating disorders. A systematic review published by Priesterroth et al. [12] reported findings on the associations between diabetes technology and diabetes-specific eating disorders in T1DM, suggesting that the increased flexibility of insulin administration with CSII may offer clinical benefits in diabetes-specific eating disorders. In a retrospective study by Pinhas-Hamiel et al. [20], sixty-three adolescent girls with type 1 diabetes were evaluated, among whom fifteen were identified with diabetes-specific eating disorders, including eight who were using CSII. Similarly, they reported that CSII is a feasible treatment option for adolescents with type 1 diabetes and coexisting disordered eating behaviors. In a large cohort study by Reinehr et al. [21], based on follow-up data from 31,556 women with type 1 diabetes, eating disorders were more frequent in girls with T1DM who were not using CSII, suggesting a potential protective effect of pump therapy. Moreover, Markowitz et al. [22] performed a prospective study including 43 adolescents monitored over six months following the initiation of CSII therapy. This study reported a significant reduction in DEPS-R scores over time, indicating that CSII may contribute to normalize eating patterns, in turn reducing risk for the development of diabetes-specific eating disorders.

The finding of our study that DEPS-R scores were significantly lower in the CSII group within the poor glycemic control group (HbA1c > 9%) suggests a potential protective effect of insulin pump therapy against diabetes-specific disordered eating. Despite suboptimal metabolic control, children and adolescents using CSII may benefit from the psychological and behavioral advantages of this modality, such as increased treatment flexibility, reduced insulin omission behaviors, and a decreased sense of dietary restriction. These factors could contribute to a lower risk of developing diabetes-specific disordered eating. This observation aligns with previous studies indicating that advanced diabetes technologies may positively influence psychosocial outcomes, even in the absence of optimal glycemic control.

In the study by Salah et al. [23], disordered eating behavior (DEB) was assessed using the Binge Eating Scale in adolescents with T1DM and it was found that no cases with DEB were observed among those in CSII treatment.

In our study, other factors that may contribute to an increased risk of eating disorders were also examined. It is well established that disordered eating behaviors are known to increase with age during childhood and adolescence, and a similar pattern has been observed in individuals with diabetes [6,8]. In contrast, our study did not demonstrate a significant correlation between age and DEPS-R scores. Previous studies have shown that females are at higher risk for eating disorders both during childhood and in adulthood [16,24]. Consistent with these findings, our study also revealed that girls were at greater risk and the majority of participants identified as high-risk according to the DEPS-R questionnaire were female. In contrast, a number of studies have reported no significant differences in median DEPS-R scores between females and males [5,8,13]. In a meta-analysis of 45 studies examining the prevalence of eating disorder symptoms in individuals with insulin-dependent diabetes, Niemelä et al. [25] found that the pooled prevalence of eating disorder symptoms was higher in studies with a higher female prevalence. These findings suggest that gender is a significant factor in the risk of developing eating disorder symptoms, highlighting the need for assessing female patients.

In line with the literature, our findings also showed that the DEPS-R score was significantly correlated with BMI [8,13,24,26,27]. Markowitz et al. [22], in their study including 43 adolescents aged 10–17 years using insulin pump therapy, reported that participants who were overweight or obese had significantly higher DEPS-R scores compared to those with normal weight. This supports the notion that increased body weight may be a risk factor for disordered eating behaviors in youth with T1DM. Our study also demonstrated a significant positive correlation between BMI and DEPS-R scores. Lawrence et al. [16] reported that a recent history of weight gain or loss is not necessarily indicative of the development of diabetes-specific eating disorders.

Previous studies have demonstrated a significant association between diabetes-specific disordered eating behaviors and poor glycemic control [8,16]. In our study, higher DEPS-R scores were observed in children with suboptimal HbA1c levels, suggesting that disordered eating behaviors may contribute to or result from inadequate diabetes management. This finding adds to the growing body of evidence suggesting that poorer metabolic control is linked to an increased risk of disordered eating in individuals with T1DM.

The study reported by Yahia et al. [28] revealed that diabetes duration was a significant predictor of eating disorders in their cohort. Consistent with this finding, our study also demonstrated a weak but statistically significant positive correlation between diabetes duration and DEPS-R scores, suggesting that a longer disease duration may be associated with an increased risk of disordered eating behaviors in adolescents with T1DM. The observed correlation between longer diabetes duration and increased risk of disordered eating behaviors may be explained by strict dietary control, diabetes-related distress or burnout and body dissatisfaction due to prolonged exposure to insulin therapy.

CGM systems provide detailed, time-based glycemic data that reflect real-life glucose fluctuations. In this study, we also investigated the relationship between diabetes-specific eating disorder risk, as measured by the DEPS-R, and CGM metrics in children and adolescents with type 1 diabetes. By analyzing these two data sources together, our study aimed to explore whether psychosocial factors related to eating behaviors are associated with measurable differences in glycemic control. There are limited studies on this topic. In the first study, patients in the elevated DEPS-R group had significantly higher mean sensor glucose and time spent in hyperglycemia, although not with glycemic variability, standard deviation, or time in hypoglycemia [29]. Similarly, Lawrence et al. [16] reported that disordered eating behaviors were associated with suboptimal glycemic control, as evidenced by elevated average CGM glucose, reduced time in range, higher time above range, and time in level 2 hyperglycemia, whereas time below range did not significantly differ between the groups. Rama Chandran et al. [30] reported that women with type 1 diabetes and disordered eating spent a longer time above range and time in level 2 hyperglycemia, and a lower time in range compared to those without disordered eating; further, they have higher average CGM glucose levels, with a higher variability in glucose levels.

Glycemic variability can be evaluated using continuous glucose monitoring metrics such as the CV and SD. In our study, a positive correlation was found between DEPS-R scores and CV, whereas no significant association was observed with SD. This suggests that a distinct pattern of glycemic fluctuation that may not be fully captured by SD alone. CV may better reflect the glycemic instability associated with disordered eating behaviors, potentially due to its ability to account for relative variability in glucose levels, independent of absolute glucose dispersion. In contrast to our findings, SD was higher in the group with disordered eating, while CV was not different between the groups in another study [30].

Rama Chandran et al. [30] hypothesized that hypoglycemia may be more common in individuals with disordered eating behaviors due to compensatory insulin use following binge eating episodes. However, in our study, higher DEPS-R scores were not associated with increased time below range, as measured by CGM.

This study has some limitations. First, the sample size was relatively small, particularly in the CSII group, which may limit the statistical power and generalizability of the findings. Because of the small sample size and large number of statistical tests, there is a risk of finding spurious associations. Therefore, our findings require confirmation in larger groups. Second, this study lacks long-term follow-up, further limiting the ability to establish a cause-and-effect relationship. These limitations should be considered when interpreting our findings. Future longitudinal studies are necessary to confirm these results. Third, important psychosocial factors such as anxiety, body image dissatisfaction, and other emotional or behavioral aspects were not assessed, although they may significantly influence disordered eating behaviors. We acknowledge this as an important limitation and suggest that future studies include these factors to better understand their impact on eating behaviors in children with T1DM. Another limitation of our study is that data about the duration of CSII treatment prior to DEPS-R assessment were not collected, which may have influenced both glycemic control and eating behaviors. Finally, the duration of CGM data varied among participants, potentially affecting the comparability of metrics across individuals. Therefore, further longitudinal studies are needed to determine the effect of CSII on eating disorder risk in adolescents with T1DM.

## 5. Conclusions

In conclusion, clinicians need to be aware of the risks of eating disorders in children and adolescents with T1DM. Our study showed that the treatment of CSII may have a beneficial effect on the risk of eating disorders in patients with poor glycemic control. As well, from this perspective, CSII maintains its status as a potentially beneficial therapeutic approach in diabetes management. However, due to the cross-sectional design and small sample size, further studies are needed to confirm the potential benefits of CSII in diabetes-specific eating disorders.

## Figures and Tables

**Table 1:** The auxologic and clinical characteristics of CSII and MDI group.

	All	CSII (*n*:20)	MDI (*n*:44)	*p*
Age (years)	13.32 ± 3.03	13.14 ± 3.67	13.42 ± 2.69	0.732
Female% (*n*)	56.3 (36)	65 (13)	52.2 (23)	0.341
Diabetes duration (years)	3.08 (1.85–5.02)	3.94 (2.92–5.13)	2.95 (1.36–4.85)	0.075
Age at presentation (years)	9.90 (6.68–11.90)	9.05 (5.38–11.07)	10.5 (8.25–12.05)	0.094
Weight SDS	0.29 ± 1.00	0.27 ± 1.12	0.30 ± 0.94	0.918
Height SDS	0.14 ± 0.89	−0.01 ± 1.13	0.01 ± 0.77	0.994
BMI	20.84 ± 3.72	20.37 ± 3.75	21.07 ± 3.73	0.493
BMI SDS	0.31 ± 1.03	0.28 ± 1.12	0.32 ± 1.00	0.883
HbA1c %	7.15 (6.57–8.22)	7.00 (6.42–7.87)	7.3 (6.60–8.57)	0.163
DEPS-R score	11 (7–17.75)	14 (7.5–20.5)	11 (6–17)	0.302
DEPS-R score ≥ 20 *n* (%)	15 (23.4)	6 (30)	9 (20.4)	0.403

Data are presented as mean ± SD or median (25–75th percentiles). CSII: continuous subcutaneous insulin infusion; MDI: multiple daily injection; SDS: standard deviation score; BMI: body mass index; DEPS-R: Diabetes Eating Problem Survey-Revised.

**Table 2:** Median DEPS-R scores of the subgroups.

	All	CSII	MDI	*p* Value
HbA1c < 7% (*n*:25)	9 (4–15)	14 (4–15.5)	9 (4–17)	0.565
HbA1c 7–9% (*n*:29)	11 (8–19)	11 (8–24.5)	10. 5 (7–17.5)	0.408
HbA1c > 9% (*n*:9)	19 (12–32.5)	16 (9–28)	43.5 (40–43.5)	0.036 *

Data are presented as median (25–75th percentiles). * *p* < 0.05. DEPS-R: Diabetes Eating Problem Survey-Revised; CSII: continuous subcutaneous insulin infusion; MDI: multiple daily injection.

**Table 3:** Clinical characteristics according to DEPS-R scores and insulin therapy method.

	DEPS-R < 20			DEPS-R ≥ 20		
	CSII (*n*:14)	MDI (*n*:35)	*p* Value	CSII (*n*:6)	MDI (*n*:9)	*p* Value
Female/male	9/5	16/19	0.24	4/2	7/2	0.634
Age	13.01 ± 2.72	13.31 ± 2.69	0.733	13.82 ± 5.13	13.97 ± 2.83	0.140
Age at presentation	9.00 (5.75–10.62)	10.36 (8.06–12.05)	0.150	9.45 (4.37–13.40)	11.25 (8.00–12.10)	0.728
Diabetes duration	3.66 (2.67–4.85)	3.00 (1.40–4.80)	0.280	4.19 (3.01–7.12)	2.10 (1.20–5.00)	0.203
BMI SDS	−0.05 ± 1.16	0.24 ± 1.00	0.385	0.99 ± 0.72	0.75 ± 0.96	0.256
HbA1c	6.70 (6.15–7.15)	7.20 (6.60–8.30)	0.041 *	7.60 (6.47–9.17)	8.90 (7.10–9.80)	0.354

Data are presented as mean ± SD or median (25–75th percentiles). * *p* < 0.05. DEPS-R: Diabetes Eating Problem Survey-Revised; CSII: continuous subcutaneous insulin infusion; MDI: multiple daily injection; BMI: body mass index; SDS: standard deviation score.

**Table 4:** Pearson correlation coefficients between DEPS-R scores and clinical variables.

Variable	Pearson’s r	*p* Value
Age (years)	0.185	0.146
Age at diagnosis (years)	−0.112	0.387
Duration of diabetes (years)	**0.301**	0.017 *
Weight SDS	**0.278**	0.029 *
BMI	**0.329**	0.009 *
BMI SDS	**0.356**	0.004 *
HbA1c (%)	**0.403**	0.001 *
Mean glucose	**0.470**	0.003 *
GMI	**0.471**	0.003 *
TIR (%)	**−0.0410**	0.012 *
TING (%)	−0.669	0.1
TAR (high, %)	−0.129	0.442
TAR (very high, %)	**0.508**	0.001 *
TBR (low, %)	0.202	0.224
TBR (very low, %)	−0.064	0.706
Glucose SD (mg/dL)	−0.200	0.747
CV	**0.417**	0.010 *

* *p* < 0.05 indicates statistically significant correlation. DEPS-R: Diabetes Eating Problem Survey-Revised; SDS: standard deviation score; BMI: body mass index; GMI: Glucose Management Indicator; TIR: time in range; TING: time in normoglycemia; TAR: time above range; TBR: time below range; SD: standard deviation; CV: coefficient of variation.

## Data Availability

The raw data supporting the conclusions of this article will be made available by the authors on request.
